# Efficacy and Safety of Oral Acetaminophen for Premature Infants With Patent Ductus Arteriosus: A Meta-Analysis

**DOI:** 10.3389/fphar.2021.696417

**Published:** 2022-01-18

**Authors:** Xie Zi-Yun, Zhang Ruo-lin, Xia Yue-wei, Bo Tao

**Affiliations:** ^1^ Department of Pediatrics, Division of Neonatology, The Third Xiangya Hospital, Central South University, Changsha, China; ^2^ Department of Neonatology, Nanshan Maternal and Child Health Care Hospital of Shengzhen, Shenzhen, China; ^3^ Department of Neonatology, Changsha Maternal and Child Health Care Hospital, Changsha, China

**Keywords:** oral acetaminophen, patent ductus arteriosus, premature infants, meta analysis, randomized controlled trial

## Abstract

**Objective**: To systematically review the efficacy and safety of oral Acetaminophen for premature infants with patent ductus arteriosus (PDA).

**Methods**: Databases including Ovid, EMbase, Pubmed, The Cochrane Library, Cumulative Index to Nursing and Allied Health Literature (CINHAL), China National Knowledge Infrastructure (CNKI), Chinese Biomedical Database (CBM), WanFang Data, China Science and Technology Journal Database were searched to collect the randomized controlled trials (RCTs) about Acetaminophen for premature infants with PDA from inception to January 1, 2021. Quality assessment was performed through bias risk evaluation according to the Cochrane Handbook 5.1.0, and then the homogeneous studies were analyzed using Revman 5.4 software.

**Results**: A total of 16 RCTs were included, which were divided into for four subgroups: subgroup I (oral acetaminophen vs. oral ibuprofen, 13 RCTs), subgroup II (oral acetaminophen vs. intravenous indomethacin, 1 RCT), subgroup III (oral acetaminophen *vs* intravenous ibuprofen, 1 RCT), and subgroup IV (oral acetaminophen *vs* intravenous placebo, 1 RCT). In subgroup I, There was no significant difference in the ductal closure rate after the first course of drug administration [typical relative risk (RR) 0.97, 95% confidence interval (CI) 0.90 to 1.05], the accumulated ductal closure rate after two course of treatment (RR 0.96, 95% CI 0.91–1.02), and mortality (RR 1.06, 95% CI 0.75–1.49) between treatment with oral acetaminophen versus oral ibuprofen (*p >* 0.05); compared with oral ibuprofen, oral acetaminophen was associated with a significant reduction in the incidence of gastrointestinal bleeding/stool occult blood positive (RR 0.51, 95% CI 0.32 to 0.82)and oliguria (RR 0.62, 95% CI 0.42–0.91) (*p* < 0.05).

**Conclusion**: The meta analysis approves the facts that there is no significant difference in the efficacity in premature infants with PDA between oral acetaminophen and buprofen or indometacin, but compared to ibuprofen, oral acetaminophen may decrease the incidence of oliguria and gastrointestinal bleeding. More reliable conclusions should be made through large-size, multi-center, well-designed RCTs.

## 1 Introduction

Patent ductus arteriosus (PDA) is a common complication in premature infants and has a significant impact on their potential outcome. The risk of PDA occurrence increases with decreasing gestational age (GA). PDA occurs in up to 65% of premature infants with GA <28 weeks (Bose and Laughon 2007). Epidemiological studies have shown that large-scale PDA causes severe hemodynamic changes in premature infants. Hemodynamically significant PDA (hsPDA) is intimately linked to the medical prognoses of premature infants, as it has been associated with elevated risks of mortality and intraventricular hemorrhage (IVH), bronchopulmonary dysplasia (BPD)/chronic lung disease (CLD), necrotizing enterocolitis (NEC), and other conditions (Irmesi et al., 2014). At present, pharmacological intervention remains the preferred strategy for the treatment of hsPDA in premature infants. The common drugs administered for this purpose are non-steroidal anti-inflammatory agents such as indomethacin and ibuprofen (Tekgunduz et al., 2013), both of which are non-specific cyclooxygenase (COX) inhibitors (Sivanandan and Agarwal 2016) and are associated with the risk of severe adverse reactions, such as visceral vasoconstriction, gastrointestinal bleeding (GIB) and perforations, inhibition of platelet aggregation, and renal failure (Oncel and Erdeve 2015). Therefore, the search for alternative pharmacological treatments remains clinically significant.

The action of acetaminophen on prostaglandin H2 synthetase (PGHS) occurs at a different site than those of indomethacin and ibuprofen (Anderson 2008), and its inhibition of prostaglandin (PG) synthesis is not accompanied with peripheral vasoconstriction (Graham et al., 2013). This may decrease the risk of related complications, and thus acetaminophen should theoretically be safer to use in premature infants. The use of acetaminophen as a treatment for premature infants with hsPDA has received increased attention in recent years, with a growing number of studies validating its efficacy; therefore, its potential as an alternative drug for the treatment of PDA in premature infants has become increasingly significant (Oncel and Erdeve 2015). Previous meta-analyses on the efficacy and safety of acetaminophen are limited by inconsistent selection criteria, the quality of the literature surveyed, and insufficient sample sizes, which have resulted in a lack of generalizability and replicability of their results (Terrin et al., 2016; Huang et al., 2018; Ohlsson and Shah 2020). The aim of this study was to investigate and review randomized controlled trials (RCTs), and assess the efficacy and safety of acetaminophen administration for the treatment of PDA in premature infants by using a meta-analysis approach, in order to provide clinical evidence for drug interventions for PDA in premature infants.

## 2 Materials and Methods

### 2.1 Inclusion Criteria and Exclusion Criteria

#### 2.1.1 Inclusion Criteria

1) Research object:samples were <37 weeks’ gestation premature infants. 2) Literature type: studies in international journals addressing RCTs about oral acetaminophen treatment in preterm infants with hsPDA were included, with language and country not specified. we use translation software to translate other languages except English into Chinese for data extraction. 3) Interventions: the studies concerning oral acetaminophen treatment and indomethacine/ibuprofen treatment were included. 4) Study type: clinical RCTs. 5) This systematic review and meta-analysis was created according to the Cochrane Handbook for Systematic Reviews (Intervention version) and follow the PRISAM guidelines (Liberati et al., 2009).

#### 2.1.2 Exclusion Criteria

1) RCTs with severe biases; 2) articles lacking sufficient original data; 3) articles failing to disclose outcome variables, for which data analysis could not be conducted; 4) repetition of the same experiment; and 5) summaries of expert experience, reviews, commentaries, and theoretical analyses.

### 2.2 Intervention Protocol

Intervention groups included those who were administered oral acetaminophen, whereas control groups included those who were administered ibuprofen or indomethacin, regardless of the administration method.

### 2.3 Outcome Measurements

Primary outcome variables included the ductal closure rate after the first course of drug administration, the accumulated ductal closure rate after two courses of treatment, and mortality. Secondary outcome variables included the incidence of NEC, BPD/CLD, IVH. retinopathy of prematurity (ROP), GIB/stool occult blood (OB) positivity, oliguria, serum creatinine (sCr), and alanine aminotransferase (ALT).

### 2.4 Literature Retrieval

The searched databases included the Ovid, EMbase, Pubmed, The Cochrane Library, Cumulative Index to Nursing and Allied Health Literature (CINHAL), China National Knowledge Infrastructure (CNKI), Chinese Biomedical Database (CBM), WanFang Data, China Science and Technology Journal Database, from inception until January 1, 2021. Studies referenced in our search results were also consulted to supplement relevant literature obtained from our search. The search terms used were “acetaminophen” [Title/Abstract] OR “paracetamol” [Title/Abstract] AND “patent ductus arteriosus” [Title/Abstract] OR “PDA” [Title/Abstract] (Table 1).

**TABLE 1:** Search stategy for Pubmed database.

#1 paracetamol [mh] OR paracetamol OR acetaminophen [mh] OR acetaminophen
#2 “Ductus Arteriosus, Patent” [mh] OR “Ductus Arteriosus” [mh] OR Ductus
Arteriosus OR “patent ductus arteriosus” OR PDA
#3 (“infant, newborn” [mh] OR newborn OR neonate OR neonatal OR premature
OR low birth weight OR VLBW OR LBW or infan* or neonat*) NOT (animals [mh]
NOT humans [mh])
#4 randomized controlled trial [pt] OR controlled clinical trial [pt] OR Clinical
Trial [ptyp] OR randomized [tiab] OR placebo [tiab] OR clinical trials as topic [mesh: noexp] OR randomly [tiab] OR trial [ti]
#5 #1 AND #2 AND #3 AND #4

### 2.5 Literature Selection and Data Extraction

Three researchers (Xie Ziyun, Xia Yuewei, Zhang Ruolin) first performed literature selection and data extraction independently, and the results were then cross-checked. Upon encountering disagreements, a fourth researcher (Bo Tao) was consulted. During the literature selection process, the titles and abstracts of the articles were first used to eliminate irrelevant articles. The full contents of the remaining papers were then perused, and the inclusion and exclusion criteria detailed above were used to determine the final selection. The extracted information mainly included the following: 1) general information on the selected study, including authorship, year of publication, country of publication; 2) nature of study: RCT; 3) general characteristics of subjects, including sample size, GA, and birth weight; 4) dosage, method, and course of drug administration; and 5) outcome indicators.

### 2.6 Bias Risk Evaluation

The three assessment researchers determined the risks of bias of the selected studies by using RCT bias risk evaluation methods (Hayden et al., 2013) as outlined in Cochrane Handbook 5.1.0, including selection bias, performance bias, attrition bias, publication bias, and other biases. The analysis results were defined as “yes” (low bias), “no” (high bias), or “unclear” (bias-related information is not clear or bias cannot be determined).

### 2.7 Statistical Analysis

The meta-analysis was conducted using RevMan 5.4.1 software (Cochrane Collaboration, 2014; http://ims.cochrane.org/revman). We reported dichotomous outcome data as relative risks with their respective 95% confidence intervals, whereas continuous variables were represented as mean differences and 95% confidence intervals, and subjected to statistical analysis. Heterogeneity was assessed using χ^2^ and *I* ^2^ tests. When the analysis results showed no heterogeneity (*p* ≥ 0.10 or *I* ^2^ < 50%), we adopted a fixed-effects model for describing potential publication bias. When the analysis results showed the presence of heterogeneity (*p* < 0.10 or *I* ^2^ ≥ 50%), we chose a random-effects model. Subgroup analysis was conducted if the heterogeneity was significant.

## 3 Results

### 3.1 Search Results

**FIGURE 1:**
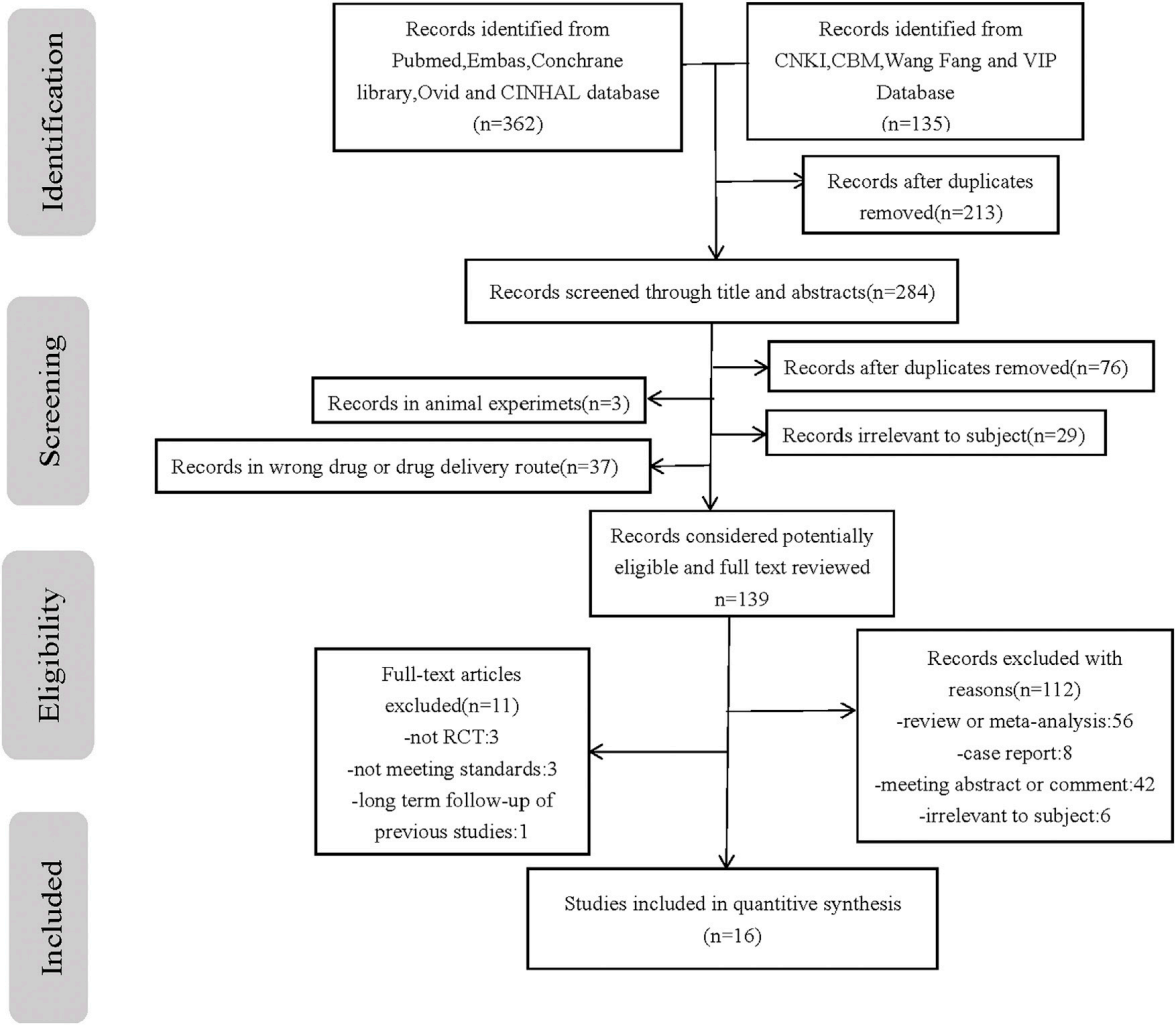
Flow chart of the study selection process. PRISMA flow diagram with process of identification, screening, eligibility and included studies in systematic review n number of studies.

Conducted according to the previously described search protocol, the first stage of our search yielded 1,056 relevant publications. Sequential filtering was performed through further perusal of titles, abstracts, or complete contents. Evaluation by using the inclusion criteria and quality assessment allowed the final selection of 16 qualifying publications (Dang et al., 2013; Oncel et al., 2014; Dash et al., 2015; Bagheri et al., 2016; Yang et al., 2016; Wu and Zhu 2017; Yang 2017; Al-Lawama et al., 2018; Hamidi et al., 2018; Zhu 2018; El-Farrash et al., 2019; Chen et al., 2019; Ghaderian et al., 2019; Kluckow et al., 2019; Balachander et al., 2020; Kumar et al., 2020), comprising 1,603 cases. A flowchart of our literature selection process and its results are presented in Figure 1.

### 3.2 Basic Features of Selected Studies

Sixteen RCTs were included in this analysis, including 1,603 cases in total, comprising 804 cases of acetaminophen administration, 731 cases of ibuprofen administration, and 39 cases of indomethacin administration (Table 2).

**TABLE 2:** The characteristic of included studies.

Included studies	Types	Sample size(T/C)	Gestational age (T/C, weeks)	Weight (T/C, gram)	Intervention time	Intervention		Outcomes
						T	C	
Dang (2013), China	RCT	80/80	31.2 ± 1.8/0 30.9 ± 2.2	1591.9 ± 348.6/ 1531.0 ± 453.5	≤postnatal 14 days	Oral paracetamol 15 mg/kg,Q6 h × 3d	Oral ibuprofen first dose 10 mg/kg, 24 h later 5 mg/kg,Qd × 2d	(1)∼(11)
Oncel (2014), Turkey	RCT	45/45	≤30/≤30	≤1250/≤1250	postnatal 48–96 h	Oral paracetamol 15 mg/kg,Q6 h × 3d	Oral ibuprofen first dose 10 mg/kg, 24 h later 5 mg/kg,Qd×2d	(1)∼(12)
Bagheri (2016), Iran	RCT	67/62	31.5 ± 2.3/ 31.7 ± 2.2	1646.3 ± 59.1/ 1642.6 ± 58.5	≤postnatal 14 days	Oral paracetamol 15 mg/kg,Q6 h × 3d	Oral ibuprofen first dose 20 mg/kg, 24 h later10 mg/kg,Qd × 2d	(1)∼(2)
Yang (2016), China	RCT	44/43	33.6 ± 2.1/ 33.4 ± 2.1	2219.0 ± 606.0/ 2091.0 ± 657.0	≤postnatal 10 days	Oral paracetamol 15 mg/kg,Q6 h × 3d	Oral ibuprofen first dose 10 mg/kg, 24 h later 5 mg/kg,Qd × 2d	(1) (4)∼(6) (9)∼(12)
Yang (2017), China	RCT	55/55	33.7 ± 2.3/ 33. 5 ± 2.2	2066.7 ± 569.2/ 2049.2 ± 563.6	≤postnatal 10 days	Oral paracetamol 15 mg/kg,Q6 h × 3d	Oral ibuprofen first dose 10 mg/kg, 24 h later 5 mg/kg,Qd × 2d	(1) (4)∼(6) (9)∼(12)
Wu (2017), China	RCT	42/42	32.1 ± 3.1/ 33.9 ± 3.2	2416.3 ± 206.2/ 2405.6 ± 215.1	≤postnatal 14 d	Oral paracetamol 16 mg/kg,Q6 h × 3d	Oral ibuprofen first dose 10 mg/kg, 24 h later 5 mg/kg,Qd × 2d	(1)(2) (4)(6) (9)∼(12)
Dash (2015), India	RCT	38/39	28.5 ± 2.7/ 28.9 ± 2.6	989 ± 299/ 1027 ± 262	postnatal 48 h	Oral paracetamol 15 mg/kg,Q6 h × 7d	Intravenous Indomethacin 0.2 mg/kg/d,Qd × 3d	(1) (3)∼(9)
Al-Lawama (2017), Jordan	RCT	13/9	23-32/25-35	1059 ± 386 1192 ± 269	Postnatal 3d–5 d	Oral paracetamol 10 mg/kg,Q6 h × 3d	Oral ibuprofen 10 mg/kg,Qd × 3d	(1)∼(8)
Zhu (2018), China	RCT	120/120	29.30 ± 2.15/ 29.21 ± 2.27	1231.2 ± 174.0/ 1244.1 ± 177.1	≤postnatal 7 d	Oral paracetamol 15 mg/kg,Q6 h × 3d	Oral ibuprofen first dose 10 mg/kg, 24 h later 5 mg/kg,Qd × 2d	(1)∼(4)(6) (8)∼(12)
El-Farrash (2019), Egypt	RCT	30/30	31.73 ± 1.98/ 30.53 ± 1.55	1.74 ± 0.47/ 1.53 ± 0.56	Postnatal 2–7 days	Oral paracetamol 15 mg/kg,Q6 h × 3d	Oral ibuprofen first dose 10 mg/kg, 24 h later 5 mg/kg,Qd × 2d	(1)∼(6)(9)(11)∼(12)
Asadpour (2018), Iran	RCT	25/25	<37/ <37	Not-descried	Not-descried	Oral paracetamol 10 mg/kg,Q6 h × 3d	Oral ibuprofen first dose 10 mg/kg, 24 h later 5 mg/kg,Qd×2d	(1) (10)∼(12)
Cheng (2019), China	RCT	62/65	29.42 ± 1.65/ 28.86 ± 2.14	1259 ± 279/ 1184 ± 248	≤postnatal 7 days	Oral paracetamol 15 mg/kg,Q6 h × 3d	Oral ibuprofen 10 mg/kg,Qd × 3d	(1)∼(6)
Kluckow (2018), Austrial	RCT	27/28	27/27.1	1004/985	≥postnatal 14 days	Oral paracetamol 25 mg/kg,then15 mg/kg, Q12h × 5d OR 15 mg/kg,Q8h × 5d	placebo	(1)(4)(5)(8)(9)
Ghaderian (2019), Iran	RCT	20/20	30.80 (1.99)/ 30.35 (2.13)	1 230.53 (1 82.1)/ 11 25.78 (200.06)	<postnatal 14 days	Oral paracetamol 15 mg/kg,Q6h × 3d	Oral ibuprofen first dose 10 mg/kg, 24 h later 5 mg/kg,Qd × 2d	(1)(2)
Balachander (2018), India	RCT	55/55	31.58 ± 2.9/ 31.54 ± 2.9	1534.8 ± 408.2/ 1513.4 ± 414.9	Postnatal 1–28 days	Oral paracetamol 15 mg/kg,Q6h × 2d	Intravenous ibuprofen first dose 10 mg/kg, 24 h later 5 mg/kg,Qd × 2d	(1)(3) (4)∼(6)(8)
Kumar (2020), India	RCT	80/81	28.7 (1.6)/ 28.7 (1.7)	1167 (249)/ 1129 (268)	≤postnatal 72 h	Oral paracetamol 15 mg/kg,Q6h × 3d	Oral ibuprofen first dose 10 mg/kg, 24 h later 5 mg/kg,Qd × 2d	(1)∼(5) (10)

Outcomes: (1) ductal closure rate after the first course of drug administration (2) the accumulated ductal closure rate after two course of treatment (3) mortality (4) NEC (5)BPD/CLD (6)IVH (7) sepsis (8)ROP (9) GIB/stool OB positive (9) Oliguria (10) Serum creatine (11) Glutamic-pyruvic transaminase.

T, test group; C, control group.

### 3.3 Evaluation of Bias in Literature

**FIGURE 2:**
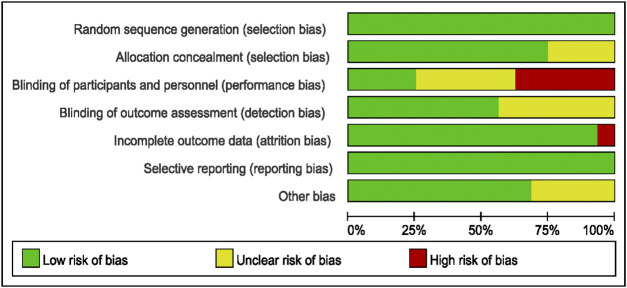
Assessment of risk of bias in randomized controlled trials.

**FIGURE 3:**
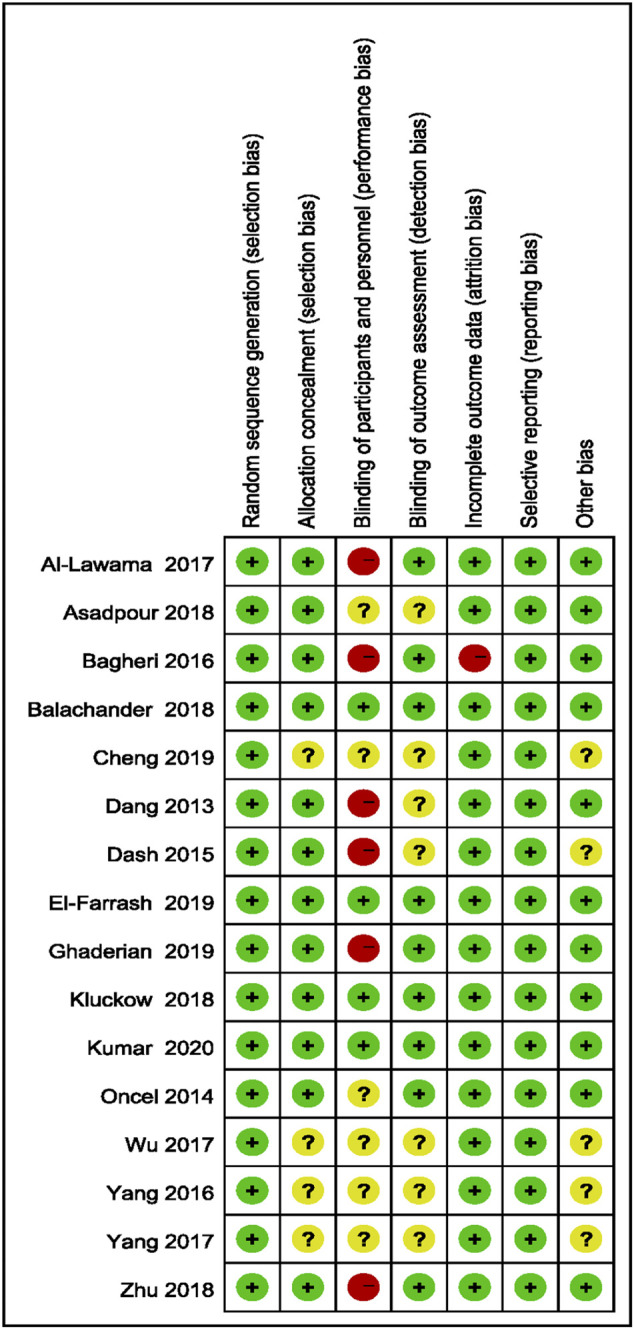
Results of the risk of bias.

Bias evaluation in this meta-analysis was performed using the Cochrane Risk of Bias Tools. The bias risks of the included studies are detailed in Figures 2, 3.

### 3.4 Results of Meta-analysis

Data were sorted into four subgroups according to intervention protocol and drug administration methods, and systematic evaluations were performed independently.

#### 3.4.1 Oral Acetaminophen vs. Oral Ibuprofen (Subgroup I)

Results from 13 RCTs were included (Dang et al., 2013; Oncel et al., 2014; Bagheri et al., 2016; Yang et al., 2016; Yang 2017; Wu and Zhu 2017; Al-Lawama et al., 2018; Hamidi et al., 2018; Zhu 2018; El-Farrash et al., 2019; Chen et al., 2019; Ghaderian et al., 2019; Kumar et al., 2020), comprising 684 cases of oral acetaminophen administration and 676 cases of oral ibuprofen administration.

##### 3.4.1.1 Primary Outcomes

**FIGURE 4:**
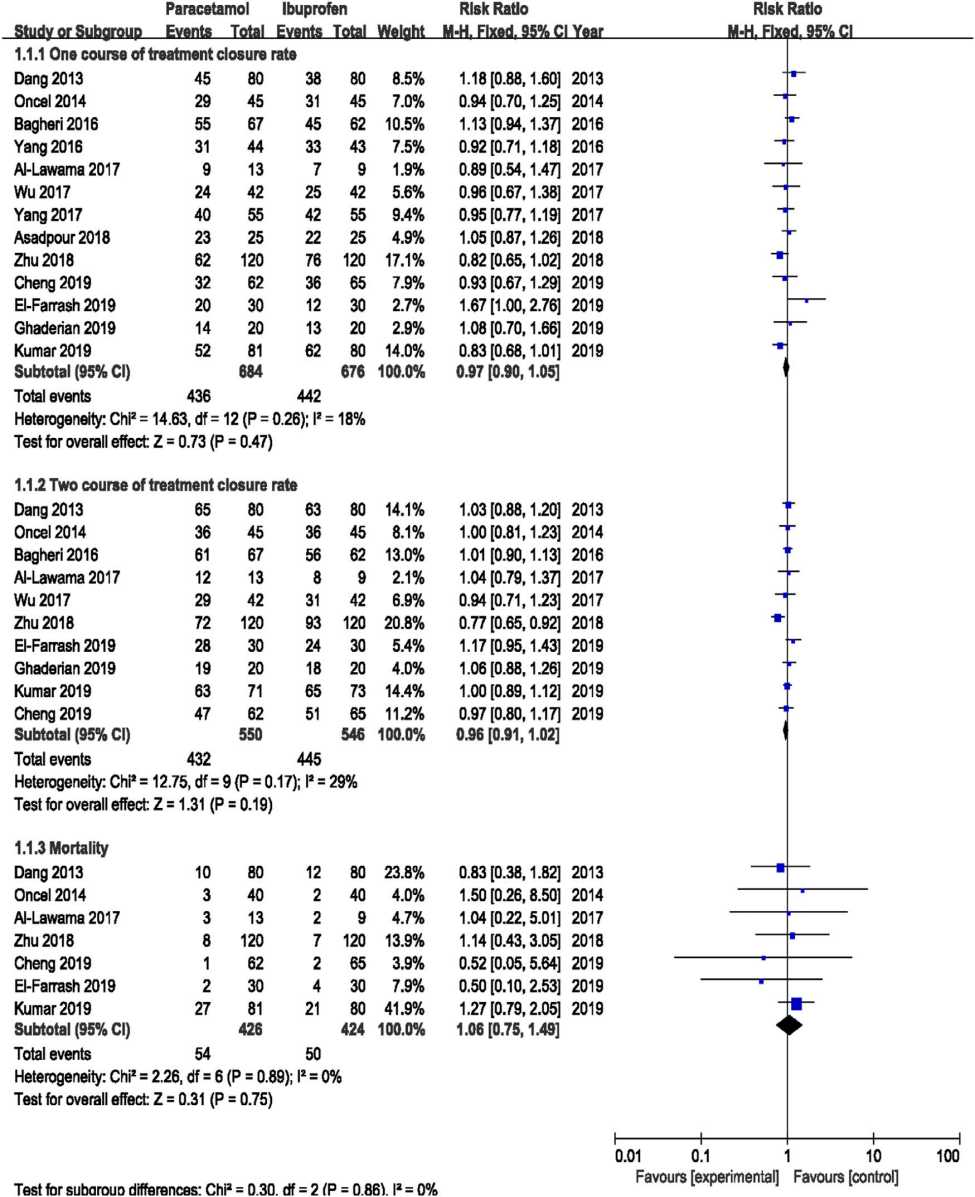
Forest plot for primary outcomes of PDA.

Meta-analysis was conducted using a random-effects model. The results showed no significant difference in the ductal closure rate after the first course of drug administration, in the accumulated ductal closure rate after two courses of treatment, and in mortality between treatment with oral acetaminophen versus oral ibuprofen (*p* > 0.05) (Table 3 and Figure 4).

**TABLE 3:** The outcomes of meta-analysis in subgroups.

Subgroup	Outcomes	RCTs	RR/MD (95%CI)	*I* ^*2*^	*p*
I	The ductal closure rate after the first course of drug administration	13	0.97 (0.90, 1.05)	18%	0.47
	The accumulated ductal closure after two courses of treatment	10	0.96 (0.91, 1.02)	29%	0.19
	Mortality	7	1.06 (0.75, 1.49)	0%	0.75
	NEC	10	1.07 (0.74, 1.56)	0%	0.71
	BPD/CLD	8	1.02 (0.76, 1.37)	0%	0.88
	IVH	9	1.03 (0.82, 1.29)	0%	0.79
	Sepsis	3	0.93 (0.64, 1.34)	0%	0.69
	ROP	4	1.06 (0.76, 1.47)	0%	0.73
	Serum creatine	8	−0.50 (−2.13, 1.13)	0%	0.55
	Glutamic-pyruvic transaminase	7	0.49 (−0.18, 1.16)	5%	0.15
	GIB/stool OB positive	7	0.51 (0.32, 0.82)	0%	0.006
	Oliguria	8	0.62 (0.42, 0.91)	23%	0.01
II	The ductal closure rate after the first course of drug administration	1	1.06 (0.96, 1.16)	NA	0.25
	The accumulated ductal closure after two courses of treatment	0			
	Mortality	1	1.03 (0.43, 2.46)	NA	0.95
	NEC	1	0.51 (0.10, 2.64)	NA	0.42
	BPD/CLD	1	0.91 (0.24, 3.40)	NA	0.89
	ROP	1	0.95 (0.77, 1.19)	NA	0.68
	GIB/stool OB positive	1	1.47 (0.62, 3.45)	NA	0.38
III	The ductal closure rate after the first course of drug administration	1	0.98 (0.79, 1.21)	NA	0.82
	Mortality	1	1.09 (0.53, 2.26)	NA	0.81
	NEC	1	1.25 (0.65, 2.42)	NA	0.51
	BPD/CLD	1	0.92 (0.61, 1.40)	NA	0.70
	ROP	1	1.05 (0.66, 1.67)	NA	0.85
	GIB/stool OB positive	1	1.09 (0.53, 2.26)	NA	0.81
IV	The ductal closure rate after the first course of drug administration	1	9.32 (0.53, 165.26)	NA	0.13
	NEC	1	1.04 (0.07, 15.76)	NA	0.98
	BPD/CLD	1	0.14 (0.01, 2.70)	NA	0.19
	ROP	1	3.11 (0.34, 28.09)	NA	0.31
	GIB/stool OB positive	1	1.04 (0.07, 15.76)	NA	0.98

NEC, necrotizing enterocolitis; BPD, bronchial pulmonary dysplasia; CLD, chronic lung disease; GIB, gastrointestinal bleeding; OB, occult blood; ROP, retinopathy of prematurity; NA,not application.

##### 3.4.1.2 Secondary Outcomes

**FIGURE 5:**
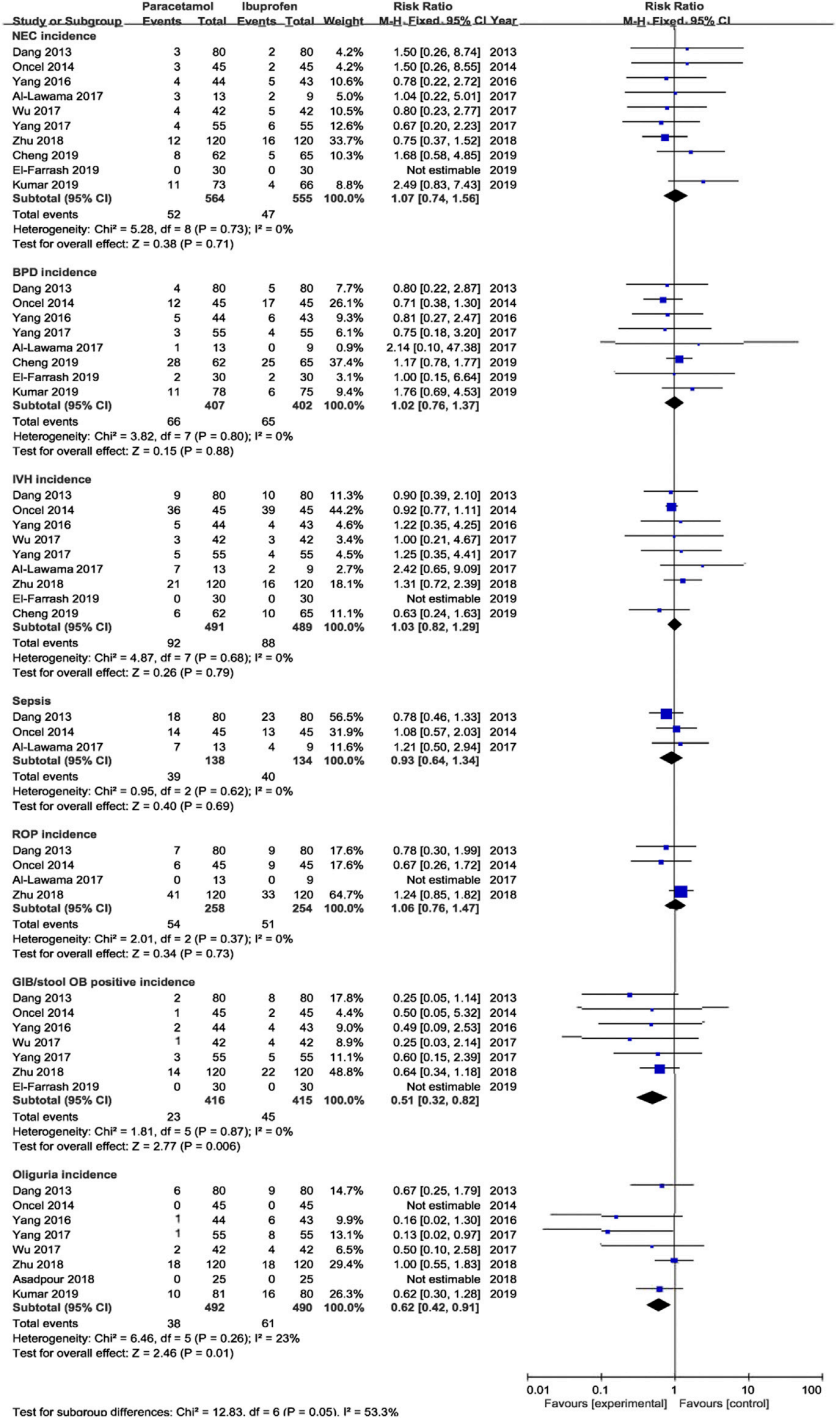
Forest plot for secondary outcomes of PDA.

**FIGURE 6:**
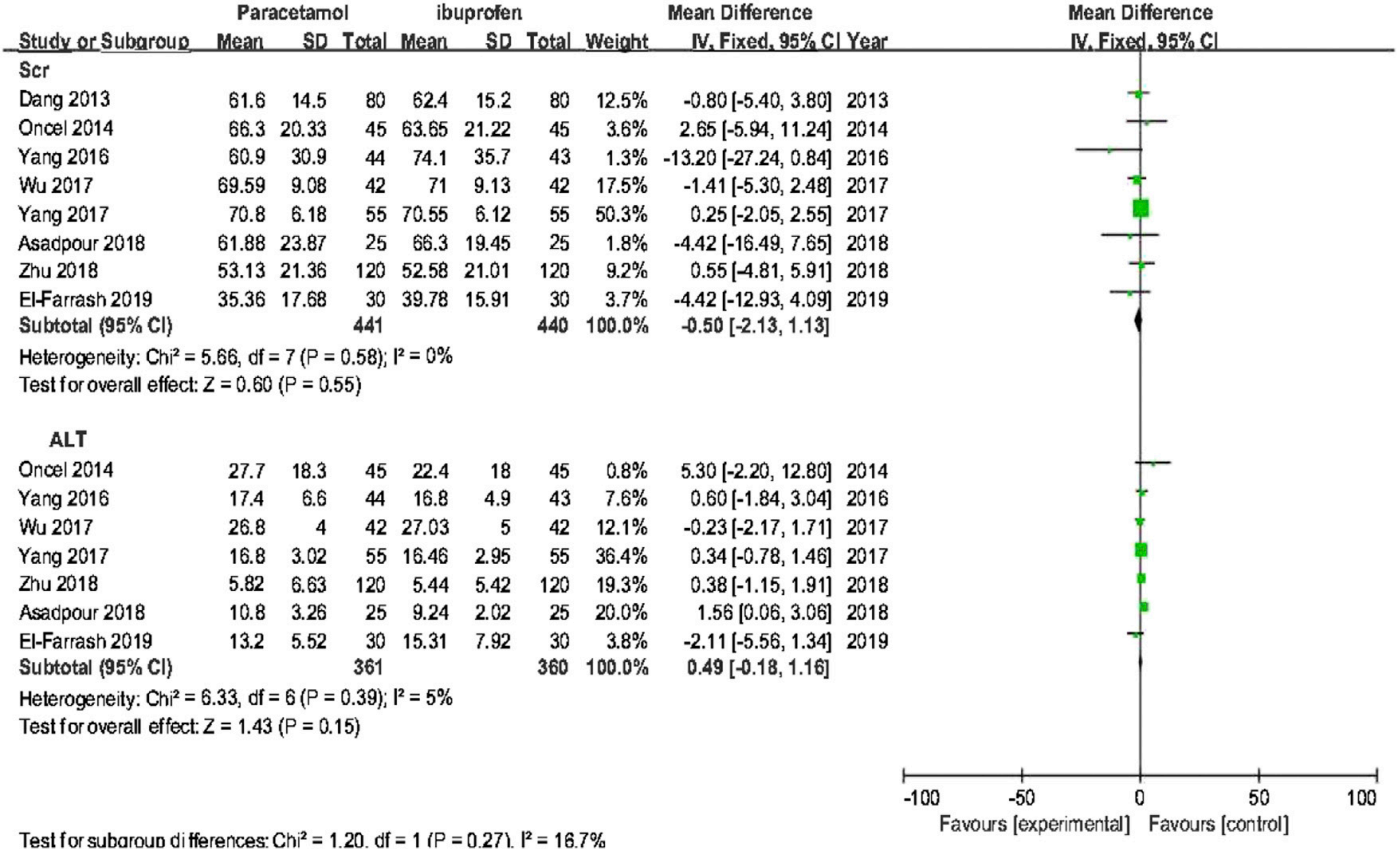
Forest plot for secondary outcomes. sCr in μmol/L; ALT in U/L.

The meta-analysis conducted using a random-effects model showed that the incidence of NEC, BPD/CLD, IVH, ROP, sCr, and ALT were not significantly different between two treatments (*p* > 0.05); oral acetaminophen caused significantly decreased rates of GIB/OB positivity and oliguria compared with oral ibuprofen (*p* < 0.05) (Table 3; Figures 5, 6).

#### 3.4.2 Oral Acetaminophen vs. Intravenous Indomethacin (Subgroup II)

**FIGURE 7:**
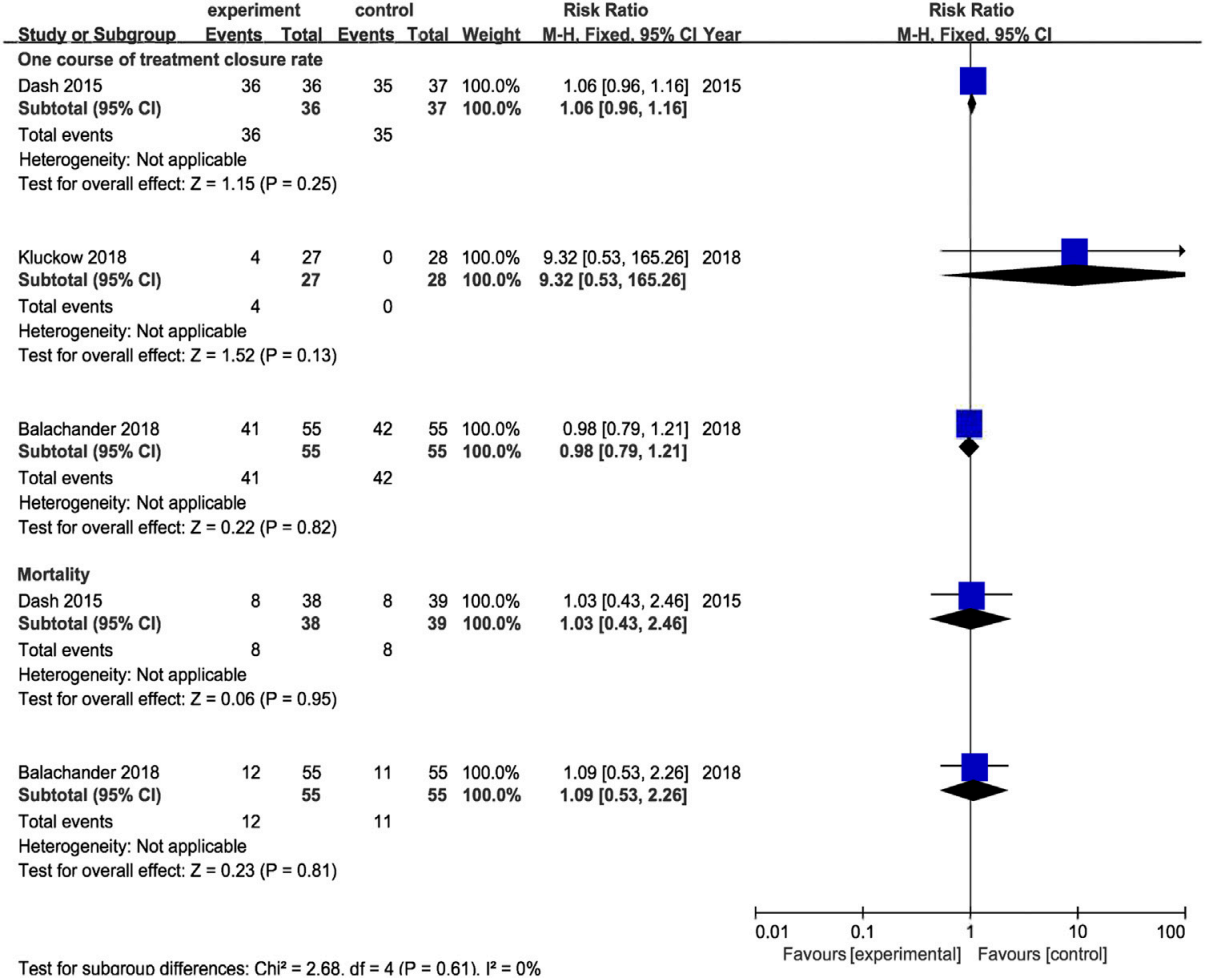
Forest plot by control (intravenous indomethacin, intravenous ibuprofen, placebo) for primary outcomes. experiment:oral acetaminphen.

Results from 1 RCT were included, comprising 38 cases of oral acetaminophen administration and 39 cases of intravenous indomethacin administration. The meta-analysis was conducted using a random-effects model. The results showed no significant difference in the ductal closure rate after the first course of drug administration, in the accumulated ductal closure rate after two courses of treatment, and in mortality, and the complication risk between the two treatments (*p* > 0.05) (Table 3 and Figure 7).

#### 3.4.3 Oral Acetaminophen vs. Intravenous Ibuprofen (Subgroup III)

Results from 1 RCT were included, comprising 55 cases of oral acetaminophen administration and 55 cases of intravenous ibuprofen administration. The meta-analysis was conducted using a random-effects model. The results showed no significant difference in the ductal closure rate after the first course of drug administration, in the accumulated ductal closure rate after two courses of treatment, and in mortality, and the complication risk between the two treatments (*p* > 0.05) (Table 3 and Figure 7).

#### 3.4.4 Oral Acetaminophen vs. Placebo (Subgroup IV)

Results from 1 RCT were included, comprising 27 cases of oral acetaminophen administration and 28 cases of placebo administration. The meta-analysis was conducted using a random-effects model. The results showed no significant difference in the ductal closure rate after the first course of drug administration, in the accumulated ductal closure rate after two courses of treatment, and in mortality, and the complication risk between the two treatments (*p* > 0.05) (Table 3 and Figure 7).

## 4 Discussion

As a consequence of underdeveloped arterial walls or abnormal PG secretion, the ductus arteriosus can remain persistently open in premature infants. PG is derived from arachidonic acid in a process involving PGHS as a key enzyme. PGHS has different sites for COX and peroxidase (POX) activities. Indomethacin and ibuprofen act on the COX site to inhibit the conversion of arachidonic acid into PGG2, decreasing PG synthesis while simultaneously affecting the production of thromboxane A2 (Anderson 2008). Thromboxane A2 acts as a vasoconstrictor, and can increase the risks of decreased visceral blood flow, impaired renal function, GIB, perforations, and other conditions (Pezzati et al., 1999; Peng and Duggan 2005; Yang and Lee 2008; Chen 2018). Acetaminophen, on the other hand, acts on the POX site to inhibit the conversion of PGG2 into PGH2 (Anderson 2008). Because of this intrinsic mechanistic difference, the use of acetaminophen may present with fewer complication risks than the use of indomethacin or ibuprofen (Le et al., 2015).

In recent years, a number of systematic evaluations have been performed for studies assessing the potential of acetaminophen as a treatment for hsPDA in premature infants; however, these evaluations remain limited by their methodology or the scope of research. Only three meta-analyses on the efficacy of acetaminophen for the treatment of hsPDA in premature infants was published respectively in 2015 and 2017, owing to limited clinical research at the time (Terrin et al., 2016; Huang et al., 2018; Ohlsson and Shah 2020). A meta-analysis published by Xiao et al. (2019) (Xiao et al., 2019). included 15 RCTs; however, its inclusion criteria limited the study to papers published in English, causing a certain risk of bias. At the same time, the study by Xiao et al. (2019) (Xiao et al., 2019). failed to account for the administration methods when categorizing treatment groups, and analyzed the results of oral and intravenous administration in conjunction. Both of these factors may have caused instabilities in the results of their analysis. In this study, inclusion and exclusion criteria were designated according to meta-analysis requirements and were used to select the 16 included studies, minimizing the risks of bias resulting from defects in the literature selection process. Subgroups were also determined according to administration methods to allow subsequent analysis.

Results from subgroup 1 in this meta-analysis showed that the use of oral acetaminophen and oral ibuprofen accounted for no significant differences in the incidences of duct closure, mortality, NEC, BPD/CLD, IVH, ROP, or septicemia, consistent with previous reports (Terrin et al., 2016; Huang et al., 2018; Ohlsson and Shah 2020). However, the incidences of GIB/OB positivity and oliguria were significantly lower in the oral acetaminophen group than in the oral ibuprofen group, suggesting that acetaminophen treatment may be safer for premature infants in some aspects. As acetaminophen is a hepatotoxic drug (Green et al., 2010), ALT was added as an outcome indicator in this study. Although elevated ALT levels were not observed to increase significantly in frequency, further clinical studies are required to assess its potential effects on liver function.

In addition, results from only a single RCT were used to reflect differences based on both intervention protocol and administration methods. Results of systemic analysis showed that when administration methods differ for different drugs, this does not account significantly for differences in the efficacy of treatments for hsPDA in premature infants. However, more RCTs will be needed to support this conclusion.

This study has some limitations. 1) Variations in durations of intervention were included in this study, which may have affected the results of the meta-analysis. 2) Many of the included studies were not blinded, whereas blinding was not specified in others. Some included studies failed to disclose whether allocation concealment was performed; therefore, the included studies may have been affected by selection bias and performance bias. 3) Systemic analysis of ALT alone was performed, whereas other parameters of liver function were disregarded, rendering an incomprehensive assessment of the hepatotoxicity of acetaminophen. 4) Only one RCT was included in subgroups II, III, and IV, which may have affected the quality of our results. García-Robles et al. (2020) are currently conducting a multicenter RCT on the effects of intravenous acetaminophen and intravenous ibuprofen, further meta-analysis of this subgroup can be conducted after the completion of the study (García-Robles et al., 2020).

In conclusion, the current evidence suggests that oral acetaminophen is similarly effective to ibuprofen and indomethacin for the treatment of premature infantile hsPDA; however, it may possess some advantages, such as decreases in the incidences of GIB and oliguria. As there is a lack of RCTs on relevant subgroups and of long-term follow-up studies at present, more multicenter large-sized RCTs, and follow-up studies will be needed to further assess the efficacy and safety of acetaminophen, especially pertaining to liver toxicity, renal toxicity, and long-term effects on the nervous system.

## Data Availability

The original contributions presented in the study are included in the article/supplementary material, further inquiries can be directed to the corresponding author.

## Abbreviations

ALT, alanine aminotransferase; BPD, bronchial pulmonary dysplasia; CLD, chronic lung disease; COX, cyclooxygenase; hsPDA, hemodynamically significant patent ductus arteriosus; IVH, intraventricular hemorrhage; NEC, necrotizing enterocolitis; GA, gestational age; GIB, gastrointestinal bleeding; OB, occult blood; PDA, patent ductus arteriosus; PG, prostaglandin; PGHS, prostaglandin H2 synthetase; POX, peroxidase; RCT, randomized controlled trial; ROP, retinopathy of prematurity; sCr, serum creatinine.
